# Antimicrobial and cytotoxicity properties of the crude extracts and fractions of *Premna resinosa* (Hochst.) Schauer (Compositae): Kenyan traditional medicinal plant

**DOI:** 10.1186/s12906-015-0811-4

**Published:** 2015-08-25

**Authors:** Sospeter Ngoci Njeru, Meshack Amos Obonyo, Samwel Onsarigo Nyambati, Silas Mwaniki Ngari

**Affiliations:** Department of Medicine, Kisii University, PO Box. 408–40200, Kisii, Kenya; Department of Biochemistry and Molecular Biology, Egerton University, P.O Box. 536–20155, Egerton, Kenya; Department of Chemistry, Egerton University, P.O Box. 536–20155, Egerton, Kenya; Present address: Fritz Lipmann Institute (FLI) – Leibniz Institute for Age Research, D-07745 Jena, Germany

**Keywords:** *Premna resinosa* (Hochst.) Schauer, Phytochemicals, Antimicrobial, Antituberculous, Herbal medicinal plant, Cytotoxicity, Bioprospecting

## Abstract

**Background:**

*Premna resinosa* (Hochst.) Schauer also called “mukarakara” in Mbeere community of Kenya is used in the management of respiratory illness. In this study we investigated antituberculous, antifungal, antibacterial activities including cytotoxicity and phytochemical constituents of this plant.

**Methods:**

Antibacterial and antifungal activities were investigated by disc diffusion and micro dilution techniques. Antituberculous activity was investigated using BACTEC MGIT 960 system while cytotoxicity was analyzed by MTT assay on Vero cells (Methanolic crude extract) and HEp-2 cells (fractions). Finally, phytochemicals were profiled using standard procedures.

**Results:**

*P. resinosa* had high antituberculous activity with a MIC of <6.25 μg/ml in ethyl acetate fraction. The antibacterial activity was high and broad spectrum, inhibiting both Gram positive and Gram negative bacteria. Dichloromethane fraction had the best antibacterial MIC of 31.25 μg/ml against Methicillin-resistant *S. aureus* while Ethyl acetate fraction had the highest zone of inhibition of 22.3 ± 0.3 against *S. aureus.* Its effects on tested fungi were moderate with petro ether fraction giving an inhibition of 10.3 ± 0.3 on *C. albicans*. The crude extract and two fractions (petro ether and methanol) were not within the acceptable toxicity limits, however dichloromethane and ethyl acetate fractions that exhibited higher activity were within the acceptable toxicity limit (CC_50_ < 90). The activity can to some extent be associated to alkaloids, flavonoids, terpenoids, anthraquinones and phenols detected in this plant extracts.

**Conclusion:**

Our findings demonstrate that *P. resinosa* has high selective potential as a source of novel lead for antituberculous, antibacterial and antifungal drugs. Of particular relevance is high activity against MRSA, *S. aureus, C. albicans* and MTB which are great public health challenge due to drug resistance development and as major sources of community and hospital based infections.

## Background

Herbal medicine, also known as practice of herbalism, or botanical medicine is the use of plants for their therapeutic and medicinal values. Historically, all medicines have an herbal origin with statistics showing that over 80 % of chemical drug compounds originated from natural material [1]. A survey by UNCTAD has shown that 33 % of drugs produced by industrialized countries are plant derived while 60 % have a natural origin [2].

Traditional herbal treatment remains the first option for patients from resource poor countries [3]. World health organization (WHO) estimates that 80 % of the world population presently use herbal medicine for some aspects of their primary health care [4]. This shift to herbal medication can be attributed to the following factors: 1. the low cost of herbal drugs endearing them with the poor mass of developing world; 2. the ‘green’ movement in the developed countries that advocates on the inherent safety and desirability of natural products; 3. the individualistic philosophy of western society that encourages self-medication, with many people preferring to treat themselves with phytomedicines [5, 6].

In developing countries like Kenya, there is an increasing attempt to incorporate traditional medicine in health care systems [7]. This is after WHO resolution in 2003 (WHA56.31) recommended the inclusion of traditional healers in management of health care. This move was to help countries document traditional medicines and remedies in their countries and to ensure their safety and efficacies is established [8]. However, it is the duty of scientists to ensure improvement and scientific justification of herbal remedies so as to allow their incorporation into health care systems as an alternative to conventional medicine.

*Premna resinosa* (Hochst.) Schauer, locally known as “Mukarakara” in Mbeere community of Embu County in Kenya, is a shrub, 1.5 - 3 m tall comprising of whitish branches and stem. The twigs have anti resistance properties, while roots are used to make perfumes and as medicine for management of respiratory related illnesses [9, 10]. However, to the best of our knowledge, there is no scientific report documented on the antituberculous properties as well as cytotoxicity properties of this plant. Lack of such information forms a major limitation in the consideration of the use of traditional herbal remedies mutually with or as an affordable alternative to conventional drugs [11]. In addition, knowledge of phytochemical constituent of plants is desirable because it can serve in identification of novel phytocompounds which can be used either in their unmodified form, as semi-synthetic compounds or as drug templates. In this study, we sought to interrogate the antimicrobial activity of water (aqueous) extract, methanol crude extract and various organic solvents fractions of root extract from *P. resinosa* as well as determine their safety by assaying for their toxicity levels.

## Methods

### Plant material

The plant for this study was identified through ethnobotanical approach. The information of its use and preparation in Mbeere community, Kenya was gleaned from local herbalist and confirmed from documentation by Relay and Brokensha [9] in *The Mbeere in Kenya (ii), Botanical identity and use*. The plant has been used for management of respiratory related illnesses. This plant is not an endangered species and it was collected in open community field and therefore no prior permission was required. The location for collection was around 0°46'27.0"S 37°40'54.9"E; −0.774156, 37.681908 of GPS co-ordinates. The identity was also confirmed by a Botanist at Egerton University where voucher specimen number NSN11 was deposited and the name checked as acceptable from [12].

### Plant Extract preparation

Root samples were chopped into small pieces of 2–3 cm and air-dried in dark at room temperature (23 ± 2 °C) to a constant weight. Using a mechanical grinder, the dried root specimens were ground to powder. The powder (50 g) was cold extracted in water with intermittent shaking to mimic the traditional local method of extraction and later lyophilized to obtain a dry powder. Another 50 g was macerated in 200 ml of methanol for 48 h. The extract was then filtered using a filter paper (whatmann 1) and the residue obtained further re-extracted using similar amount of methanol. The two volumes of filtrate were pooled together and thereafter concentrated *in vacuo* using a rotary evaporator. Afterwards, the product was allowed to air dry and the yields recorded.

Fractionation of powdered root part of *P. resinosa* was done using different solvents of increasing polarity. The root powder (50 g) was macerated in 200 ml of Petro ether with intermittent shaking for 48 h after which they were filtered using Whatman no 1 filter paper. The residue was further re-extracted using fresh solvent for 24 h and thereafter the filtrates pooled together. The resulting residue was air dried and further extracted with Dichloromethane followed by Ethyl acetate and lastly methanol using the same procedure carried out for Petroleum ether. Using a rotary evaporator, the solvent was removed from each filtrate under conditions of reduced temperature and pressure. The resulting dry extract was weighed and stored in air tight sample bottles at −20 °C until next use.

### Culturing of micro organisms

One Gram positive; *Staphylococcus aureus* (ATTC 25923) strain and Methicillin Resistant *Staphylococcus aureus* strain (clinical isolate), five Gram negative; *Escherichia coli* (ATTC 25922), *Klebsiella pneumoniae* (clinical isolate)*, Pseudomonas aeruginosa* (ATCC 27853), *Salmonella typhi* (clinical isolate) and *Shigella sonnei* (clinical isolate) and two Fungi; *Candida albicans* (ATTC 90028) and *Cryptococcus neoformans* (ATTC 66031) were investigated for antimicrobial activity. The extracts were also tested against acid fast *Mycobacterium tuberculosis* strain H37Rv (ATCC 27294). These organisms were sourced from Kenya Medical Research Institute (KEMRI) - Nairobi.

### Disc diffusion test

The antibacterial activity was assayed by disc diffusion method according to CLSI [13] and Mbaveng et al., [14] with slight modifications. Fresh inoculum was prepared by suspending activated colonies in physiological saline water (0.85 % NaCl). Using 0.5 McFarland turbidity standard, the bacteria and fungi suspensions were adjusted to 1.5 × 10^6^ CFU/ml after which they were inoculated aseptically by swapping the surfaces of the Muller Hinton (MHA) plates and sabouraud dextrose agar (SDA) plates. Whatmann filter paper (No.1) discs of 6 mm diameter were made by punching the paper, and the blank discs sterilized in the hot air oven at 160 °C for one hour. They were then impregnated with 10 μl of various stock extract solution. The methanolic and water crude extracts stock solution was at (1.0 g/ml). For fractions; petro ether, dichloromethane, and methanol fractions stock solutions were made at 500 μg/ml while ethyl acetate at 250 μg/ml. This afforded disc extract concentration of 1.0 × 10^4^ μg/disc for water and methanol crude extracts, 5 μg/disc for petro ether, dichloromethane, and methanol fractions and 2.5 μg/disc for ethyl acetate. Three standard drugs were used as positive controls: Oxacillin 10 μg/disc (Oxoid Ltd, Tokyo-Japan) and Gentamycin 10 μg/disc (Oxoid Ltd, Tokyo-Japan) for Gram positive and Gram negative bacteria respectively. Nystatin 100 μg/disc (Oxoid Ltd, Tokyo-Japan) was used as the standard drug for all fungi while discs loaded with 10 μl of DMSO was used as negative controls. The impregnated dry discs were carefully placed on the agar plates at equidistance points using a sterile forceps. A positive control as well as a negative control was incorporated in each plate and the plates incubated at 4 °C for 2 h so as to allow the extract to diffuse into the media after which they were incubated at 37 °C for 18 h. Antimicrobial activity was determined by measuring the size of the inhibition zone to the nearest mm and the results recorded. Extracts fractions that gave an inhibition zone of more than 10 mm were considered to be active [13] and therefore their MIC (Minimum inhibitory concentration) and MBC (Minimum bactericidal concentration) determined [15].

### Determination of MIC and MBC

The MIC and MBC of the plant *P. resinosa* extracts was determined for all the organisms in triplicates using broth micro-dilution assay. The petro ether, dichloromethane, and methanol fractions stock solutions were made at 500 μg/ml while ethyl acetate at 250 μg/ml with DMSO. To 100 μl of nutrient broth agar in a sterile 96 well plate, 50 μl of varying plant concentration (petro ether, dichloromethane, and methanol fractions at 500 to 3.91 μg/ml while ethyl acetate at 250 to 1.95 μg/m) was added followed by 50 μl of test organisms previously diluted to equivalent of 0.5 McFarland standard. Addition of the test organisms was done in all the wells except for wells of column 11 which contained neat DMSO and broth, this served as control to check for purity. The adequacy of the media to support the growth of the test organism was evaluated by putting the broth and the test organism in wells of column 12. The plates were then covered with a sterile “cling-on” sealer and incubated for 24 h at 37 °C. Bacterial growth was evaluated by addition of 40 μl of 0.2 mg/ml p-iodonitroterazolium chloride (INT, Sigma) to each well and incubated for 30 min. Growth of bacteria was detected by formation of a pink-red coloration while inhibition of growth was signaled by persistence of a clear coloration. The lowest concentration that exhibited color change was considered as the MIC. Minimum bactericidal concentration was determined by streaking a loopful of broth from wells that exhibited no color change onto sterile nutrient agar and sabouraud dextrose agar for bacteria and fungi respectively and thereafter incubated at 37 °C for 24 h. The lowest concentration that exhibited no growth was considered as the MBC [16].

### Antitubercular activity

The test organism *Mycobacterium tuberculosis* H37Rv ATCC 27294 was sourced from the Kenya Medical Research Institute (KEMRI), Nairobi*.* Prior to its use, the *Mycobacterium tuberculosis* was revived on Lowenstein Jensen (LJ) slants for 14 days at 37 °C following standard procedures [17, 18]. The efficacy of the plant extracts against *M. tuberculosis* was carried out using the BACTEC MGIT 960 system (BD, New York-U.S.A). This is a fully automated, high volume, non-radiometric instrument that offers continuous monitoring of culture growth. The dry crude extract (water and methanolic) was first dissolved in DMSO to a final concentration of 1 g/ml for preliminary screening. Growth supplement (0.8 ml) containing a mixture of OADC- Oleic Acid, Bovine Albumen, Dextrose and Catalase was added to five 7 ml BBL™ MGIT™ tube labeled GC (growth control), STR (streptomycin), INH (isonaizid), RIF (rifampicin), EMB (ethambutol) to provide essential substrates for rapid growth of *Mycobacteria*. 100 μl of BBL™ MGIT™ SIRE (streptomycin, isonaizid, rifampicin, ethambutol) prepared aseptically according to the manufacturers’ instruction was added to corresponding labeled BBL™ MGIT™ tube followed by addition of 0.5 ml of 1 % *Mycobacterium* suspension. *Mycobacterium* suspension was prepared by pipetting 0.1 ml Middlebrook 7H9 Broth containing *Mycobacterium* adjusted to 0.5 McFarland standard into 10 ml sterile saline aseptically. The BACTEC MGIT™ 960 system (BD, New York-U.S.A) was then loaded following the manufacturer’s instructions and incubated at 37 °C. Streptomycin at 1.0 μg/ml, isoniazid at 0.1 μg/ml, rifampicin at 1.0 μg/ml and ethambutol at 5.0 μg/ml served as the positive controls whereas DMSO was used as a negative control. The procedure was repeated using plant crude extracts at 1.0 g/ml in place of SIRE. The process was also repeated with petro ether, dichloromethane, ethyl acetate and methanol solvent fractions. The fractions were tested at concentrations ranging from 50 to 6.25 μg/ml (petro ether, dichloromethane and methanol) or 25 to 3.125 μg/ml (ethyl acetate) to determine the MIC.

### Cytotoxicity screening

MTT [3-(4, 5-dimethylthiazol-2-yl)-2, 5-diphenyltetrazolium bromide] assay was used to determine the toxicity of the extracts obtained from the plant. This is a colorimetric assay hinged on the ability of mitochondrial enzyme (Succinate Dehydrogenase) to reduce yellow water soluble MTT to an insoluble colored substance, formazan, which is spectrophotometrically measurable. The level of formazan is directly proportional to the measure of cell viability because only metabolically active cells can reduce MTT. Test cell lines were Vero cells from African green Monkey Kidney cells (*Cercopithecus aethiops* epithelial cell line; ATCC CCL-81) against methanol crude extract and HEp-2 cells (human laryngeal carcinoma cell line ATCC CCL-23) against fractions that were used as test cells for this study. The test cells were grown in growth media comprising of 100 ml DMEM, 10 ml Fetal Bovine Serum (FBS), 1 ml Penstrep, 1 ml Amphotericin B, 1 ml L Glutamine. The test cells were incubated at 37 °C in 5 % CO_2_ until they attained confluency after which they were passaged by adding 2 ml of 0.25 % trypsin and further incubated at room temperature until the cell were detached. Growth media (6 ml) was introduced to the test cells to inactivate trypsin off action, while the cell crumps formed were broken gently by sucking and releasing the cell suspension using a pipette. 2 ml of the harvested cells were then transferred into a 50 ml vial and topped up to 50 ml mark using growth media. A cell suspension of 100 μl (1 × 10^5^ cell/ml) was seeded into two rows of wells A-H in a 96-well micro-titer plate for one sample. The test cells were then incubated in 100 μl growth media at 37 °C and 5 % CO_2_ for 48 h to form a confluent monolayer. The growth medium was then aspirated off and replaced with 100 μl of maintenance medium comprising of 100 ml DMEM, 2 ml Fetal Bovine Serum (FBS), 1 ml Penstrep, 1 ml Amphotericin B, 1 ml L-Glutamate and 0.1 ml Gentamycin. Afterwards, cells were exposed to increasing concentrations of respective plant extracts (from 1.95 μg/ml to 500 μg/ml) and incubated at 37 °C for 48 h. This was followed by a further incubation period of 4 h in 10 μl of 5 mg/ml MTT solution after aspirating off the plant extracts. This was followed by addition of 100 μl acidified isopropanol (0.04 N HCl in isopropanol). The well plate was gently shaken for 5 minutes to dissolve the formazan and then optical density measured using ELISA Scanning Multiwell Spectrophotometer (Multiskan Ex labssystems) at 562 nm and 690 nm as reference. Rows of cells containing medium without plant extracts were included to act as negative control. Cell viability (%) was calculated at each concentration as follows using the formula [19].

$$ \mathrm{Cell}\ \mathrm{viability}\ \left(\%\right) = \frac{\left({\mathrm{OD}}_{\mathrm{sample}562}-{\mathrm{OD}}_{690}\right)}{{\mathrm{OD}}_{\mathrm{control}562}-{\mathrm{OD}}_{690}}\times 100 $$

### Phytochemical tests

Phytochemical tests were done to determine the class of compounds present in the active fractions that could be responsible for activity and/or cytotoxicity. They were identified by characteristic colour changes based on standard procedures as described previously [8, 20–23]. The results were reported as (+) for presence, and (−) for absence.

### Alkaloids

Six to eight drops of Dragendorf reagent was mixed with 2 ml of the extract. Formation of brownish-red precipitate indicated presence of alkaloids. The Dragendorf reagent was prepared by mixing two reagents: reagent 1 and reagent 2 in equal parts. Reagent 1 was made by dissolving 8.5 g of Bismuth subnitrate in a solution of 10 ml acetic acid and 40 ml of distilled water while as Reagent 2 was prepared by dissolving 8 g of potassium iodide in 20 ml of water [22, 23].

### Phenols

Phenols were detected using ferric ferichloride which was prepared by dissolving 0.1 g of ferric ferichloride in 10 ml of water. Equal volumes (2 ml) of both ferric ferichoride and the plant extract were mixed. Formation of a violet- blue color or greenish color was evidence that phenols presences [22, 23].

### Terpenoids

1 gram of Vanillin was mixed with 100 ml of concentrated sulphuric acid after which 2 mls of the resultant solution was mixed with 2 mls of the plant extract. Formation of a blue- green ring or pink- purple coloration signified presences of terpenoids [22, 23].

### Anthraquinones

0.5 ml of the plant extract was mixed with 0. 5 ml of 10 % methanolic potasium hydroxide. Red coloration indicated presences of anthroquinones. 10 % methanolic potassium hydroxide was prepared by dissolving 0.5 g of potassium hydroxide pellets in 50 mls of methanol [22, 23].

### Flavonoids

5 ml of dilute aqueous ammonia solution was added to a portion of the aqueous filtrate of the plant extract, followed by concentrated sulphuric acid. A positive test result was confirmed by the formation of a yellow coloration that disappeared instantly [20, 21].

### Statistical analysis

Ms Excel 2010 data sheets and Graphpad Prism version 6 were used to analyze the data. The data on cytotoxicity was expressed as a percentage of the untreated controls. CC_50_ values, which is the concentration that kills 50 % of the test cells, was determined by Regression Analysis. A particular fraction’s extract was considered cytotoxic if it had CC_50_ of less than 90 μg/ml [24]. In addition, unpaired student’s t-test was used to test for statistical significance in the differences between the treatments and the control in this study. A *p* value of less than 0.05 was considered to indicate statistical significance. Values were expressed as mean ± S.E.M.

## Results and discussion

The information on use and preparation of *P. resinosa* plant in Mbeere community-Kenya was gleaned from tradipractitioners and herbalist and confirmed from documentation by Relay and Brokensha [9]. The plant has been used traditionally for management of respiratory related illnesses. Since the traditional preparation involved steeping the roots peels in water, alcoholic beverage, or chewing root peels, we first screened for antimicrobial activity using water and methanolic crude extract to mimic this traditional extraction mode. Although herbal practice in many places as well as in Mbeere community involves utilization of water as the main herbal extraction solvent, studies have shown that methanol organic solvent is much better and potent [5, 25, 26], a fact corroborated by antimicrobial results of the present study (Tables 1 and 2). This could be attributed to polarity of methanolic solvent that confers the ability to extract a variety of bioactive molecules (Table 3). Polarity of the solvent also influences the qualitative and quantitative composition of the active compounds sequestered into herbal extract(s). This could partly explain the higher activity demonstrated by methanolic crude extract compared to water extract [5, 25–27].

**Table 1:** Antimicrobial activity for the *P. resinosa* crude root extract

Extract	Diameter of zone of inhibition in mm		
	Organism		
	EC	SA	CA
WT	7.3 ± 1.3	7.0 ± 0.6	11.7 ± 2.0
MOH	8.7 ± 1.3	11.7 ± 0.3	11.0 ± 0
PC	22 ± 0	24.7 ± 1.3	16.3 ± 0.9
NC	0	0	0

*WT* Water crude extract at 1.0 x 10^4^ μg, *MOH* Methanol crude extract at 1.0 x 10^4^ μg, *EC E. coli*, *SA S. aureus*, *CA C. albicans*, *PC* Positive control (Oxacillin 10 μg/disc and Gentamycin 10 μg/disc for Gram positive and Gram negative bacteria respectively. Nystatin 100 μg/disc for fungi), *NC* Negative control (Discs loaded with 10 μl of DMSO), *n* = 3, *Values* mean ± SEM

**Table 2:** Antituberculous activity of *P. resinosa* crude extract

Sample	Solvent	GU	R/S
*Premna resinosa* (Hochst.) Schauer	WT	400	R
	MOH	0	S
	SIRE	0	S
	GC	400	R
	NC	400	R

*WT* Water crude extract at 1 g/ml, *MOH* Methanol crude extract at 1 g/ml, *SIRE* Positive control of streptomycin at 1.0 μg/ml, isonaizid at 0.5 μg/ml, rifampicin at 1.0 μg/ml and ethambutol at 5.0 μg/ml, *GC* Growth control, *NC* Negative control of media treated with DMSO, *R* Resistant, *S* Sensitive

**Table 3:** Phytochemical tests for *P. resinosa* methanolic crude extract

Phytochemicals	Methanol extract result
V-T	++++
A-F	+++
MK-A	++++
D-A	+++
F-P	+++

*V-T* Vanillin test for terpenoids, *A-F* Ammonia test for Flavonoids, *MK-A* Methanolic Potassium hydroxide test for Anthraquenones, *D-A* Dragendorff test for Alkaloids, *F-P* Ferric Chloride test for Phenols, *−* Absent phytochemicals, *+* Low concentration of phytochemicals,*++* Medium concentration, *+++* High concentration of phytochemicals

The methanolic crude extract yield was 1 g (Table 4) and hence we began our antimicrobial screening with a high concentrations of 1.0 × 10^4^ μg/disc. Both aqueous and methanol crude extracts had broad spectrum activity, inhibiting the growth of Gram positive, Gram negative bacteria and fungi at 1.0 × 10^4^ μg/disc (Table 1). The highest inhibition in water extract was 11.7 ± 2.0 for *C. albicans* while the highest inhibition in methanolic extract was 11.7 ± 0.3 for *S. aureus*. The activity against *E. coli* and *C. albican* in both extracts was not statistically different at p = 0.67 and p = 0.77 respectively. However methanolic crude extract had higher activity against *S. aureus* compared to water crude extract and the difference in activity between the two extracts was statistically significant (p = 0.02). Additionally, the positive control drugs in all cases had higher activity than the plant crude extract. We also did fractionation using organic solvents of increasing polarity. Each fraction was tested on a panel of microorganisms. The results varied with the extract fraction used for testing. This may suggest that the root part of *P. resinosa* contains several antibacterial and antifungal compounds of different polarities as supported by phytochemical studies (Table 5). Fractionation enhanced antibacterial activities in all tested cases compared to crude methanolic extracts (Table 6). For example, ethyl acetate fraction had a zone of inhibition of 22.3 ± 0.3 against *S. aureus* while the crude methanolic extract had an inhibition zone of 11.7 ± 0.3 for the same organism. This may imply that, there is higher sequestration of active principle(s) at certain level of polarity explaining the high but varied antibacterial activity demonstrated by fractions from solvents of different polarities. It is also suggestive that, there is possibility of antagonism of various antibacterial active compounds when lumped together as in crude extracts [28], thus explaining for low antibacterial activity in crude extracts. This is therefore indicative that, fractions are the best candidates for the treatment of diseases associated with the tested microorganisms than crude extracts. Nevertheless, some extracts fails to have enhanced activity on fractionation as exhibited by antifungal activity in this study. This may imply either that, the antifungal principles act together in a synergistic manner and that is why crude methanolic extract had higher antifungal activity, or that, fractionation had dilution effect on the antifungal principle(s) thus explaining diminished antifungal activity with fractionation (Tables 1 and 6) [28].

**Table 4:** *P. resinosa* extracts yield

		Yields in grams					
		Crude extract		Fractions			
Sample	Family	MOH	WT	PE	DCM	EA	MOH
*P. resinosa*r	Compositae	1	NR	<1	<1	2.531	1

*WT* Water, *MOH* Methanol, *PE* Petro ether, *DCM* Dichloromethane, *EA* Ethyl acetate, *MOH* Methanol, *NR* Not recorded

**Table 5:** Phytochemicals of *P. resinosa* fractions

Extract fraction	V-Ts	A-F	MK-A	D-A	F-P
Petro ether	-	-	++++	+++	-
Dichloromethane	++++	++++	++++	++++	++++
Ethyl acetate	+++	++++	+++	++	+++
Methanol	++++	++++	+++	-	++++

*V-T* Vanillin test for terpenoids, *A-F* Ammonia test for Flavonoids, *MK-A* Methanolic Potassium hydroxide test for Anthraquenones, *D-A* Dragendorff test for Alkaloids, *F-P* Ferric Chloride test for Phenols, *−* Absent phytochemicals, *+* Low concentration of phytochemicals, *++* Medium concentration, *+++* High concentration of phytochemicals

**Table 6:** Antimicrobial activity for *P. resinosa* fractions

Fractions	Diameter of zone of inhibition in mm								
	Microorganism								
	Gram positive		Gram negative					Fungi	
	SA	MRSA	PA	EC	KP	SH	ST	CA	CR
PE	18 ± 0.6	NT	0	11.3 ± 0.3	0	0	0	10.3 ± 0.3	NT
DCM	19.3 ± 0.3	10.6 ± 0.6	0	10.3 ± 0.3	0	9.7 ± 0.7	0	9.3 ± 0.3	0
EA	22.3 ± 0.3	0	0	0	NT	11.7 ± 0.3	0	6.3 ± 0.3	7.3 ± 0.3
MOH	19.0 ± 0.6	10.7 ± 0.9	0	11.7 ± 0.3	7.3 ± 0.3	12.3 ± 0.3	7.0 ± 0.6	10.0 ± 0.6	NT
PC	33.7 ± 0.3	24.3 ± 0.3	23.7 ± 0.6	17 ± 0.6	15.7 ± 0.3	19.7 ± 0.6	21.3 ± 0.3	16.3 ± 0.3	20.3 ± 0.3
NC	0	0	0	0	0	0	0	0	0

*PE* Petro ether fraction at 5 μg/disc, *DCM* Dichloromethane fraction at 5 μg/disc, *EA* Ethyl acetate fraction at 2.5 μg/disc, *MOH* Methanol fraction at 5 μg/disc, *PA P. aerogenosa*, *EC E. coli*, *SA S. aureus*, *KP K. pneumoniae*, *MRSA Methicillin Resistant S. aureus*, *SH Shigella*, *ST S. typhi*, *CA C. albicans*, *CR Cryptococcus*, *PC* Positive control (Oxacillin 10 μg/disc and Gentamycin 10 μg/disc for Gram positive and Gram negative bacteria respectively. Nystatin 100 μg/disc for fungi), *NC* Negative control (Discs loaded with 10 μl of DMSO), *n* = 3, *values* Mean ± SEM

The lowest MIC of 31.25 μg/ml was recorded in petro ether and methanolic fraction against *S. aureus*. The dichloromethane fraction had MIC of 31.25 μg/ml against methicillin resistant *S. aureus* (MRSA) and it was also cidal with MBC of 125 μg/ml. The latter case is more interesting considering that the MIC/MBC ratio is 4 suggesting the killing/cidal effect could be expected (Table 7). Additionally, out of 20 tested cases for MIC, 8 (40 %) had MIC of less than 100 μg/ml; the set threshold for plant extract [28]. Generally, activity against Gram positive bacteria was higher than Gram negative strains and fungi. This is in agreement with previous studies that plant extracts are more active against Gram positive bacteria than Gram negative bacteria. The higher sensitivity of Gram-positive bacteria could be attributed to their cell wall topology which has outer peptidoglycan layer which is not an effective permeability barrier as compared to the outer phospholipid membranes of Gram-negative bacteria [25, 29, 30]. Difference in sensibility was also evidence among tested strains in both crude and fraction extracts. This could be due to genetic differences between different strains. This proofs the necessity of antibiogram prior to prescription as a precautionary measure in mitigating drug resistance development [31].

**Table 7:** MIC and MBC for the *P. resinosa* fractions

	MIC in μg/ml				
Fraction	MRSA	SA	EC	SH	CA
PE	NT	31.25	62.5	NT	NT
DCM	31.25	-	-	NT	NT
EA	NT	62.5	NT	-	NT
MOH	125	31.25	62.5	62.5	62.5
	MBC in μg/ml				
PE	NT	static	static	NT	NT
DCM	125	static	static	NT	NT
EA	NT	Static	NT	static	NT
MOH	500	static	static	static	500

*PE* Petro ether fraction, *DCM* Dichloromethane fraction, *EA* Ethyl acetate fraction, *MOH* Methanol fraction, *EC E. coli*, *SA S. aureus*, *MRSA Methicillin Resistant S. aureus*, *SH Shigella*, *ST S. typhi*, *CA C. albicans*, *NT* Not tested, *−* >500 μg/ml

We also investigated the antituberculous activity of crude extracts and fractions (Tables 2 and 8). Methanolic crude extract (at 1 g/ml) was highly active in inhibiting tubercle growth. We went further and investigated activity of organic solvent fractions with view of determining the MIC. Interestingly, all fractions had varying level of growth inhibition in a concentration dependent manner. The highest activity was by ethyl acetate fraction (MIC <6.25 μg/ml) and dichloromethane (MIC <12.5 μg/ml). The MIC for petro ether fraction was 25.0 μg/ml while that for methanolic fraction was 50.0 μg/ml. This depicts high potential of the extract fractions (especially ethyl acetate fraction considering the threshold MIC level of 100 μg/ml [28]) to be tapped for novel drug lead for management and/or treatment of TB. This is supported by our data showing that the level of inhibition by drug standards (SIRE) (GU- 0) is same as that achieved by some of our tests samples (Table 8). But it is also important to stress that, our fractions were loaded with various bioactive compounds as shown in Table 5. It is possible that the active compound could be only a small proportion of the fractions’ extract and maybe if purified, it could be even of lower amount than the standard drugs.

**Table 8:** Antituberculous activity for *P. resinosa* fractions

Antituberculous testing				
Plant	Fraction	Concentration μg/ml	GU	R/S
*Premna resinosa* (Hochst.) Schauer	PE	50.0	0	S
		25.0	0	S
		12.5	400	R
		NC	400	R
		SIRE	0	S
	DCM	50.0	0	S
		25.0	0	S
		12.5	0	S
		NC	400	R
		SIRE	0	S
	EA	25.0	0	S
		12.5	0	S
		6.25	0	S
		NC	400	R
		SIRE	0	S
	MOH	50.0	0	S
		25.0	400	R
		12.5	400	R
		NC	400	R
		SIRE	0	S

*PE* Petro ether fraction, *DCM* Dichloromethane fraction, *EA* Ethyl acetate fraction, *MOH* Methanol fraction, *SIRE* Positive control of streptomycin at 1.0 μg/ml, isonaizid at 0.5 μg/ml, rifampicin at 1.0 μg/ml and ethambutol at 5.0 μg/ml, *GU* Growth unit, *NC* Negative control of media treated with DMSO, *R* Resistant, *S* Sensitive

Since in some instances people chew the roots of the plant *P. resinosa* for herbal treatment, we also sought to answer the question whether *P. resinosa* extracts are cytotoxic. The methanolic crude extract (CC_50_ of 1.26 μg/ml), Petro ether fraction (CC_50_ of 1.88 μg/ml) and methanolic fractions (CC_50_ 4.78 μg/ml) were all not within the acceptable toxicity limit (CC_50_ > 90.00 μg/ml) (Table 9). This can however be viewed as “a double edged sword”. In one way, it is bad for a drug as it will be lethal to subjects. Nevertheless, structural modification can be undertaken with view of improving on their safety [19]. On the other hand, this can be good news since the fractions can be tapped as candidates for anti-cancerous drugs [5], especially now that their lethality was toward cancerous human cell lines.

**Table 9:** Cytotoxicity of *P. resinosa* in μg/ml

	CMOH	PE	DCM	EA	MOH
CC_50_ (μg/ml)	1.26	1.88	96.87	>500	4.78

*CMOH* Crude methanolic extract, *PE* Petro ether fraction extract, *DCM* Dichloromethane fraction extract, *EA* Ethyl acetate fraction extract, *MOH* Methanol fraction extract, *CC*_*50*_ Concentration that kills 50 % of the cells (Vero cells with CMOH and HEp-2 cells with fractions)

The observed bioactivity and/or cytotoxicity could be attributed to the array of phytochemicals that tested positive in the sample extracts. These included alkaloids, flavonoids, phenols, terpenoids and anthraquinones. Bioactive molecules are usually found accumulated as secondary metabolites in various parts of the plant and at different concentrations [32]. In this regard, the root is one of the major depository sites of such compounds making it a chief part for herbal bioprospecting. Flavonoids which tested positive in all samples except in petro ether fraction have general antibacterial activity. They have been shown to work by complexing and altering the conformation(s) of microbial proteins thus inactivating microbial enzymes and interfering with bacterial cell wall adhesins. In a similar fashion as terpenoids (also present in all samples except petro ether fraction), they also mediate their antibacterial activity through microbial membrane disruption [32]. Other studies have associated flavonoids with antituberculous activity and it is believed that their mode of action is by inhibiting various pathways in *Mycobacteria* including *de novo* biosynthesis of fatty acid, inhibiting mycolic acid biosynthesis, proteosome inhibition, topoisomerase inhibition, inhibition of phosphatidyl-inositol 3-kinase, induction of cell cycle arrests, accumulation of p53 or enhanced expression of c-fos and c-myc genes [18, 33]. In addition, recent studies done by [34] indicated that phenolic compounds have antimycobacterial properties although their mode of action is not well known. Similarly, studies have established that alkaloids have both antibacterial and antifungal activities, especially against *S. aureus* and *C. albicans* [35]. The results of this study provide to some extent scientific rationalization of the possible therapeutic use of *P. resinosa* plant in traditional medicine, and also confirms the impact of ethnopharmacological approach when investigating plants for their bioactivity [31].

## Conclusion

A major outcome of the current study is the identification of the ethyl acetate and dichloromethane fractions which yielded the best antituberculous activity (MIC of <12.5 and <6.25 μg/ml respectively) as well as the highest antibacterial activity (with zones of inhibition of 19.3 ± 0.3 and 22.3 ± 0.3 respectively and the lowest antibacterial MIC of 31.25 μg/ml), all within the acceptable toxicity limit (CC50 > 90 μg/ml). Our findings also demonstrates for the first time and to the best of our knowledge that, *P. resinosa* plant has very high selective potential as a source of novel lead for antituberculous, antibacterial and antifungal drugs. Of particular importance is its high activity against MRSA, *S. aureus, C. albicans* and MTB which are currently posing great public health challenge due to drug resistance development and as major sources of community and hospital based infections. Indeed, more work is needed to identify the specific active ingredients, with a view of deciphering the mode(s) of action of the plant compounds. Additionally, the information of cytotoxicity opens a new frontier to pursue in search of novel antineoplastic compounds.
